# Hand, hip and knee osteoarthritis in a Norwegian population-based study - The MUST protocol

**DOI:** 10.1186/1471-2474-14-201

**Published:** 2013-07-05

**Authors:** Nina Østerås, May Arna Risberg, Tore K Kvien, Lars Engebretsen, Lars Nordsletten, Dag Bruusgaard, Unni-Berit Schjervheim, Ida K Haugen, Hilde Berner Hammer, Sella Provan, Britt Elin Øiestad, Anne Grete Semb, Silvia Rollefstad, Kåre Birger Hagen, Till Uhlig, Barbara Slatkowsky-Christensen, Ingvild Kjeken, Gunnar Flugsrud, Margreth Grotle, Sølve Sesseng, Hanne Edvardsen, Bård Natvig

**Affiliations:** 1 National Resource, Center for rehabilitation in Rheumatology, Department of Rheumatology, Diakonhjemmet Hospital, Oslo, Norway; 2 Norwegian Research Center for Active Rehabilitation Department of Orthopaedic Surgery, Oslo University Hospital, Oslo, Norway; 3 Department of Rheumatology, Diakonhjemmet Hospital, Oslo, Norway; 4 Department of Orthopaedic Surgery, Oslo University Hospital Ullevål, Oslo, Norway; 5 Department of General Practice, Institute of Health and Society, University of Oslo, Oslo, Norway; 6 Primary Health Care, Ullensaker Municipality, Akershus, Norway; 7 Department of Radiology, Diakonhjemmet Hospital, Oslo, Norway

**Keywords:** Osteoarthritis, Hand, Hip, Knee, Observational study, Study protocol

## Abstract

**Background:**

Knowledge about the prevalence and consequences of osteoarthritis (OA) in the Norwegian population is limited. This study has been designed to gain a greater understanding of musculoskeletal pain in the general population with a focus on clinically and radiologically confirmed OA, as well as risk factors, consequences, and management of OA.

**Methods/Design:**

The Musculoskeletal pain in Ullensaker STudy (MUST) has been designed as an observational study comprising a population-based postal survey and a comprehensive clinical examination of a sub-sample with self-reported OA (MUST OA cohort). All inhabitants in Ullensaker municipality, Norway, aged 40 to 79 years receive the initial population-based postal survey questionnaire with questions about life style, general health, musculoskeletal pain, self-reported OA, comorbidities, health care utilisation, medication use, and functional ability. Participants who self-report OA in their hip, knee and/or hand joints are asked to attend a comprehensive clinical examination at Diakonhjemmet Hospital, Oslo, including a comprehensive medical examination, performance-based functional tests, different imaging modalities, cardiovascular assessment, blood and urine samples, and a number of patient-reported questionnaires including five OA disease specific instruments. Data will be merged with six national data registries. A subsample of those who receive the questionnaire has previously participated in postal surveys conducted in 1990, 1994, and 2004 with data on musculoskeletal pain and functional ability in addition to demographic characteristics and a number of health related factors. This subsample constitutes a population based cohort with 20 years follow-up.

**Discussion:**

This protocol describes the design of an observational population-based study that will involve the collection of data from a postal survey on musculoskeletal pain, and a comprehensive clinical examination on those with self-reported hand, hip and/or knee OA. These data, in addition to data from national registries, will provide unique insights into clinically and radiologically confirmed OA with respect to risk factors, consequences, and management.

## Background

Osteoarthritis (OA) is currently the most prevalent joint disease and is one of the core diseases in the Bone and Joint Decade 2011-2020 [1]. It can occur in any joint, but is most commonly seen in selected joints of the hand, spine, and lower limb. Clinically, OA is characterised by joint pain, crepitus, swelling, stiffness, restricted range of motion, fatigue, and functional limitations. OA is one of the leading causes of pain and disability for the adult population worldwide [2] and may have considerable personal and societal consequences in relation to health problems [3], work disability [4], and economical costs [5-7]. Recently, sequela of the OA disease was defined as one of the major contributors to years lived in less than ideal health (years lived with disability (YLDs)) [8]. The burden of OA is better described for the knee and hip, than for the hand joints and generalised OA [9].

OA is strongly age-related - it is uncommon before the age of 40, but the prevalence rises rapidly with age thereafter. Women are affected more frequently among those aged > 45 years [10], and the gender difference is most prominent for hand and knee OA [2]. Prevalence estimates vary, as the estimate is dependent on the joint of interest, the method of assessment, and the disease definition used [11]. In a US cohort of individuals at least 45 years of age, 16% had symptomatic knee OA and 10% had symptomatic hip OA [12,13]. The prevalence of symptomatic hand OA in the Framingham OA study was 16% and 8% for women and men, respectively [14]. With an aging population and the current epidemic of obesity, a strong risk factor for OA [15], the OA prevalence is expected to increase in the coming years [15].

OA has traditionally been defined on the basis of radiographic features only, or on a combination of radiographic features and joint symptoms [11]. A number of clinical criteria and radiographic classification systems have been established to promote uniformity in the reporting of OA definitions (i.e. The American College of Rheumatology (ACR) criteria [16-18], the Kellgren-Lawrence scale [19], and the Osteoarthritis Research Society International (OARSI) score [20]). Recently, a distinction between structural joint changes (‘OA disease’) and individuals’ self-reported symptoms of OA (‘OA illness’) was recommended for treatment effect evaluations, which may also be useful in trial design and recruitment [21].

Self-reported OA is sometimes used as a practical alternative in gaining epidemiological knowledge about OA, and self-reported physician diagnosed arthritis was found to be the question with the highest accuracy in prediction of radiologically confirmed OA [22]. The association between the prevalence of self-reported OA and the prevalence of criteria-based diagnoses of OA has been investigated to a limited degree for hip and knee [23-28], but not for hand OA. Although self-reported OA fails to reach complete accuracy, previous studies have found satisfactory sensitivity and specificity and recommend this approach as a screening tool in large population-based studies [23,26-28]. However, conflicting results have also been published [25], and for complete ascertainment procedure in clinical trials, a clinical examination and radiographs may still remain necessary [27].

### Imaging modalities

While late-stage OA is often characterised by both demonstrable structural changes (loss of joint space, osteophytes and changes in the subchondral bone) and patient-reported joint pain, stiffness and disability, there are only weak correlations between symptoms and OA pathology, particularly in early stages of the disease [21]. It has been estimated that 40-80% of individuals with radiographic changes have concurrent symptomatic OA disease [14,29]. The discrepancy between imaging and clinical findings is becoming more complex with increased use of sensitive imaging techniques such as magnetic resonance imaging (MRI), which more frequently demonstrates structural abnormalities, inflammatory changes, and degeneration of soft tissue compared to conventional radiographs [30]. Most studies using MRI have included patients with knee OA, showing that bone marrow lesions and synovitis are associated with pain [31]. Similar results have been found in hand OA [30,32], although more studies are needed. Ultrasound also has the ability to demonstrate both changes in bone and soft tissue, and a scoring system for use in hand OA has been developed [33,34], but further work to validate ultrasound features of OA is needed [35].

### Clinically relevant phenotypes

Recently, there has been an interest in classifying OA populations into different clinical and/or structural phenotypes, which might improve the understanding of the disease and allow the treatment to become more targeted and tailored. A proposal for differentiation of clinical phenotypes based on age and primary causative features has been published [15]. Further, cluster analyses using data from knee OA participants in the Osteoarthritis Initiative Study have been performed to identify phenotypes with different clinical outcomes [36]. More research on classifications of different phenotypes may further improve the development within diagnosis, treatment, and monitoring of the OA disease.

### Hand OA

Hand photography is an easy and inexpensive imaging method involving no harm for the subjects. A standardised procedure for taking digital hand photographs, a set of reference photographs, and standardised reading procedure for diagnosing hand OA has been published showing adequate inter- and intraobserver variation [37]. However, the diagnostic value of hand photography in hand OA needs further investigation. The importance of measuring aesthetic damage in individuals with hand OA has been recognised [38,39]. The extent to which deformities in affected joints may have a negative influence on individuals’ social life has not been systematically assessed. The relation between hand OA and hypermobility is scarcely investigated, and previous research has shown conflicting evidence [9]. More insight into this association would be interesting in order to provide preventive strategies. Obesity has been shown to be an important risk factor for OA, especially for knee OA [2] and for total hip replacement [40,41], but there is now also increasing evidence suggesting a relationship between obesity and hand OA [9]. Whether OA is part of a “metabolic syndrome” including overweight, hypertension, and diabetes is being debated and needs further examination. Affection of the carpometacarpal joint in the thumb may compromise function in the whole hand, and was found to contribute more to pain and disability than affection of the interphalangeal joints [42]. More research on factors associated with OA in the carpometacarpal joint in the thumb is needed.

### Cardiovascular morbidity

Recently, there has been substantial interest in the relationship between various arthritic disorders in relation to cardiovascular morbidity [43]. Some studies have shown an association between OA and cardiovascular morbidity and mortality [44], and it has been suggested that atherosclerosis may contribute to the initiation or progression of OA [45,46]. An association between arterial stiffness and hand OA has been found, but the significant individual relationship was largely attributable to the confounding effect of age [43]. These findings and novel hypotheses need to be explored in longitudinal studies.

### Quality of care

Several international recommendations and standards of care for OA management have been developed [2,47-51]. However, there has been limited focus on evaluation of the quality of care provided for OA patients, and previous studies have revealed low adherence with published recommendations [52-54], and have suggested that OA care was suboptimal [55-58]. An English study by Steel et al. showed that the pass rate for quality indicators on OA management was only 29% [59], and we expect that the situation in Norway is similar. The only Norwegian study in this area is a prospective study on physiotherapy performance in patients with knee OA, in which the authors concluded that there is a need to promote high quality evidence into physiotherapy practice [60].

### Sick leave and health care utilisation

In Sweden, it was reported that individuals with knee OA have close to twofold increased risk of sick leave and about 40-50% increased risk of disability pension compared to the general population [61]. It is unknown whether similar figures can be found in Norway. The costs of OA care will be heavily influenced by the trends in OA occurrence, the severity of the disease, and the consequences of OA in function and work ability. More research is warranted on utilisation of self-management and treatment in primary, secondary, or tertiary care.

### Physical activity

At least 30 minutes physical activity per day for at least 3 days a week is recommended as a general guideline for those with knee and hip OA [62]. Knowledge on type and level of physical activity in individuals with OA is limited. A large survey conducted in the US indicated that those with OA were less active than healthy individuals [63], but the activity level according to affected joint was not investigated. A recent US study showed that only 13% of men and 8% of women with knee OA met the aerobic component of the 2008 Physical Activity Guidelines for Americans (≥150 minutes/week of moderate-to-vigorous–intensity activity lasting ≥10 minutes) [64]. A recent Norwegian study with accelerometer-determined physical activity revealed that only one in five adults or older people met the current national physical activity recommendations to accumulate at least 30 minutes of daily moderate intensity physical activity [65]. The study also showed that overweight and obese participants performed less overall physical activity compared to normal weight participants [66]. However, knowledge of physical activity levels in Norwegian individuals with OA is needed.

### The purpose of this study

This study has been designed to recruit individuals from an unselected adult population to gain more knowledge about clinically and radiologically confirmed OA, risk factors, consequences, and management of OA. The study incorporates different imaging techniques (conventional radiographs, MRI, and US), patient-reported outcomes and performance-based outcome measures, cardiovascular morbidity and metabolic syndrome in individuals with hand, hip and/or knee OA. Such data will be valuable for various epidemiological and clinical studies.

The main aims of the present study are to evaluate:

1. The prevalence of hand, hip, and knee OA in a population-based cohort.

2. The association between structural abnormalities (conventional radiographs, MRI and US) and symptoms in individuals with hand, hip, and knee OA.

3. The concordance between OA features in the hip and knee joints identified by conventional radiographs and US examination.

4. The associations between different OA phenotype classifications and clinical outcomes.

5. The associations between cardiovascular morbidity as well as vascular biomarkers and OA phenotypes.

6. The associations between OA in the carpometacarpal joint in the thumb and self-reported symptoms and function.

7. The frequency of self-reported concerns related to aesthetic damage in the joints of the hand and of self-reported participation restrictions due to aesthetic damage.

8. Obesity as a risk factor for hand OA, and associations between different OA locations and medical conditions in a metabolic syndrome (defined as overweight, hypertension, and diabetic disease).

9. The quality of OA care, health care utilisation, and sickness absence among those with hand, hip, and knee OA.

10. The association between self-reported physical activity, function, and pain in individuals with hand, hip, and knee OA.

## Methods/Design

### Design and setting

The MUST has been designed as an observational study which includes a population-based postal survey (Q1) as well as a subsample (the MUST OA cohort) who attend a comprehensive clinical examination (CE) and complete OA specific questionnaires (Q2 and Q3).

The target population for the postal population-based survey is the inhabitants in the Ullensaker Municipality, which is a rural community 40 kilometres northeast of Oslo. Some of the nearly 30 000 inhabitants commute to Oslo for work, but for the last 10 to 15 years, the work opportunities and the number of Ullensaker inhabitants have expanded due to the building and the subsequent management related to the national Oslo Airport Gardermoen. This development has resulted in some minor changes in demographic factors, as many persons of younger age and with higher education levels have moved into the municipality.

### Participants in the population-based survey

All inhabitants aged between 40 and 79 years receive the postal four page questionnaire (Q1). Information on residence is provided by the Population Register. The questionnaire is returned by mail in a pre-paid envelope. One written reminder is sent to non-responders after eight weeks.

### Participants in the comprehensive clinical examination

Individuals eligible for the comprehensive clinical examination are identified using the self-reported OA item in the population-based postal survey (Q1): ‘Have you ever been diagnosed with osteoarthritis in hip/knee/hand by a medical doctor and/or x-ray?’ Response categories include: Yes, hip/ Yes, knee/ Yes, hand/ No. Those who self-report OA in their hip, knee, and/or hand, will receive a postal request to attend a clinical examination (CE) performed at Diakonhjemmet Hospital, Oslo. Two written reminders are sent to non-responders. The participants are screened for clinical examination eligibility by the project coordinator, and those who are unable to walk (with or without walking aids) and/or do not speak or understand Norwegian language are excluded for the clinical examination.

### Assessments

#### The population-based postal survey questionnaire (Q1)

The four-page questionnaire includes demographic questions as well as questions about life style, general health, musculoskeletal pain, OA, comorbidities, health care utilisation, medication use, and functional ability [67-70]. The questionnaire includes items on self-reported cardiovascular disease and symptoms (Table 1).

**Table 1:** The population-based postal survey questionnaire (Q1)

**Measure**	**Measurement scale**
**Demographic variables**	
Gender	Female/male
Age	Birth year
Marital status	Married, cohabitating/Separated, divorced/Widowed/single
Body height	Centimetres
Body weight	Kilograms
Employment status	Working full time/working part time/not working/student/working full time in the home/unemployed or seeking work/age retired/disability pension/sick leave
Education	Lower secondary school/ Higher secondary school/University 1-4 years/University >4 years
**Lifestyle variables**	
Frequency of leisure time physical activity [67]	Never/Less than once a week/Once a week/2-3 times a week/Almost every day
Intensity of leisure time physical activity [67]	I take it easy without breaking into sweat or losing my breath/I push myself so hard that I lose my breath and break into a sweat/I push myself to near-exhaustion
Duration of leisure time physical activity [67]	Less than 15 minutes/16-30 minutes/30 minutes-1 hour/More than 1 hour
Daily smoking	Yes/no
**Musculoskeletal pain and symptoms**	
Standardised Nordic Questionnaire (SNQ) Pain in past year [68]	Body manikin showing 10 body parts: Yes/No
SNQ Pain affected daily activities [68]	Body manikin showing 10 body parts: Yes/No
SNQ Pain in past 7 days [68]	Body manikin showing 10 body parts: Yes/No
Average musculoskeletal pain past 7 days	NRS: 0-10
Osteoarthritis diagnosis	‘Have you ever been diagnosed with osteoarthritis in hip/knee/hand by a medical doctor and/or x-ray?’ Response categories include: Yes, hip/ Yes, knee/ Yes, hand/ No.
Most troublesome OA joint	Knee/Hip/Hand
**Health, comorbidity, and subjective health complaints**	
General health nowadays	Poor/Not so good/Good/Very Good
Heart disease	Yes/No
Lung disease	Yes/No
Cancer	Yes/No
Diabetes	Yes/No
Osteoporosis	Yes/No
Irregular heartbeat	Yes/No
Chest pain	Yes/No
Breathing difficulties	Yes/No
Gastrointestinal symptoms	Yes/No
Skin problems	Yes/No
Tiredness/fatigue	Yes/No
Dizziness	Yes/No
Anxiety	Yes/No
Depression	Yes/No
**Health care utilization**	
Medical doctor	Number of visits past year
Medical specialist	Number of visits past year
Physiotherapist	Number of visits past year
Chiropractor	Number of visits past year
Occupational therapist	Number of visits past year
Home nurse	Number of visits past year
Alternative therapy	Number of visits past year
Hospital admissions	Number of days past year
**Medication use**	
Glucosamine	Yes, daily/Yes, sometimes/No
Paracetamol	Yes, daily/Yes, sometimes/No
Anti-inflammatory medication	Yes, daily/Yes, sometimes/No
Use this medication due to musculoskeletal pain	Yes, daily/Yes, sometimes/No/Do not know
**Functional ability**	
10-ADL Multidimensional Health Assessment Questionnaire (MDHAQ) [69]	0-40; Without any difficulty/With some difficulty/With much difficulty/Unable to do
COOP/WONCA Physical fitness [70]	Very heavy activity/Heavy /Moderate/ Light/Very light
COOP/WONCA Feelings [70]	Not at all/Slightly/Moderately/Quite a bit/Extremely
COOP/WONCA Daily activities [70]	No difficulty at all/A little bit of difficulty/Some difficulty/Much difficulty/Could not do
COOP/WONCA Social activities [70]	Not at all/Slightly/Moderately/Quite a bit/Extremely

#### The OA specific questionnaire (Q2)

Participants who are eligible and scheduled for the clinical examination fill in the OA specific questionnaire (Q2) at home before attending the clinical examination. This questionnaire includes standardised instruments targeting health related quality of life, physical activity, cardiovascular disease questions, and OA disease specific instruments [71-79] (Table 2).

**Table 2:** Measures in the OA specific questionnaire (Q2)

**Measure**	**Measurement scale**
**Quality of life**	
SF-36 Vitality [71]	0-100
SF-36 Physical functioning [71]	0-100
SF-36 Bodily pain [71]	0-100
SF-36 General health perceptions [71]	0-100
SF-36 Physical role functioning [71]	0-100
SF-36 Emotional role functioning [71]	0-100
SF-36 Social role functioning [71]	0-100
SF-36 Mental health [71]	0-100
EuroQol EQ-5D [72]	(-0.59-1)
**Physical activity**	
International Physical Activity Questionnaire (IPAQ) vigorous-intensity activity [73]	Mean minutes/week, MET-minutes/week
IPAQ moderate-intensity activity [73]	Mean minutes/week, MET-minutes/week
IPAQ walking [73]	Mean minutes/week, MET-minutes/week
IPAQ sitting [73]	Mean minutes/week, MET-minutes/week
Physical activity with increased heart rate and breathing for at least 30 min	1-5; 3 times a week/1-2 times a week/1-2 times a month/Do not do regular physical activity/Unable to do due to reduced physical functioning
**Osteoarthritis specific questionnaires**	
Knee injury and Osteoarthritis Outcome Score (KOOS) pain [74]	0-40 or 0-100 (normalised score)
KOOS symptoms [74]	0-20 or 0-100
KOOS ADL [74]	0-68 or 0-100
KOOS Sport/Rec [74]	0-16 or 0-100
KOOS QOL [74]	0-16 or 0-100
Hip disability and Osteoarthritis Outcome Score (HOOS) pain [75]	0-36 or 0-100
HOOS symptoms [75]	0-28 or 0-100
HOOS ADL [75]	0-68 or 0-100
HOOS Sport/Rec [75]	0-16 or 0-100
HOOS QOL [75]	0-16 or 0-100
Australian/Canadian Osteoarthritis Hand Index (AUSCAN) pain [76]	0-20
AUSCAN stiffness [76]	0-4
AUSCAN function [76]	0-36
AUSCAN total [76]	0-60
Functional Index for Hand Osteoarthritis (FIHOA) [77]	0-30
Michigan Hand Outcomes Questionnaire (MHOQ) aesthetics [78]	0-40
**Patient-reported quality of osteoarthritis care**	
OsteoArthritis Quality Indicator (OA-QI) questionnaire [79]	Yes/No/Do not remember, not overweight, no such problems, no pain/discomfort, not severely troubled
**Cardiovascular sypmtoms/disease**	
Paroxystic atrial fibrillation, chronic atrial fibrillation, heart failure, dyspnea, peripheral edema, angina pectoris, chest pain (description, location, time points, frequency, duration), use of nitroglycerine, acute myocardial infarction (AMI), heart surgery (percutaneus coronary intervention (PCI), coronary artery bypass grafting (CABG), heart valve replacement), peripheral vascular disease, cerebral haemorrhage, cerebral infarction, transitory ischemic attach (TIA), diphtheria, scarlet fever, rheumatic fever, family history of premature cardiovascular disease in first degree relatives, age at heart infarction, sudden death in family for unknown reason	Yes/No
**Other diseases**	
High blood pressure (or antihypertensive treatment), diabetes, metabolic disease, kidney disease, hepatic disease, gastric ulcer, ulcerous colitis, Crohn's disease, deep vein thrombosis, lung embolia, iridocyclitis	Yes/No
**Immunological diseases**	
List any other immunological disease (e.g rheumatoid arthritis, ankylosing spondylitis, psoriasis, psoriatic arthritis, Sjøgren's disease, polymyalgia rheumatica, systemic lupus erythematosus (SLE), Lichen ruber, vitiligo etc)	List of comorbidity
**List all current medication use**	Medication list
**Other**	
Smoking	Never/Daily/stopped smoking more than 6 months ago
Alcohol use	Daily/Weekly/Monthly/Never
Age at menopause	Age
Oestrogen use	Yes/no
Previous use of contraception pills	Yes/no
Number of children	Number

#### The comprehensive clinical examination (CE)

The comprehensive clinical examinations are scheduled on a weekly basis with eight or nine participants at any one session. These examinations are performed by physicians and health professionals in the project group, radiographers at Diakonhjemmet Hospital, and trained medical students. The clinical examination lasts for about four hours; transportation time not included. Beforehand, the participants receive a postal, OA specific questionnaire (Q2) to fill in at home (Table 2). They answer some additional questions on pain and OA symptoms (Q3) during the clinical examination (Table 3). The clinical examination (CE) includes a wide array of assessments (Table 4):

1) The medical assessment.

The medical assessments are performed by a medical doctor (BSC) with many years of clinical experience in rheumatology and by trained final-year medical students under supervision of BSC. The medical assessment will include an assessment of clinical signs of OA as well as knee joint effusion using the Stroke test [80]. Verification of the ACR criteria for hip, knee and hand OA are performed [16-18]. The medical doctor also scores ‘The Doctor Global assessment of OA disease’, and asks when the participants were diagnosed with OA and when they first experienced OA symptoms. Further, the doctor investigates familial OA, dominant hand and foot, and registers current (and former) employment status according to level 0-5 on the Tegner Activity Scale [81].

2) Conventional radiographs.

Bilateral radiographs of the hand, hip, and knee joints are performed on all participants at the Diakonhjemmet Hospital according to a standardised protocol. Hand: Posterior-anterior projection of hands. Hip: Supine lying with a calibration bullet between the thighs. Anterior-posterior projection of the total pelvic frontal view. Knee: The procedure is performed using the SynaFlexer™ frame which standardises knee flexion angle to 20° and external foot rotation to 5°, as described by Kothari et al. [82]. In this protocol, a 10° caudal beam angulation ensures alignment of the beam with the medial tibial plateau (fluoroscopy). X-ray views include anterior-posterior, lateral, and patella tangential. The radiographs are scored according to the Kellgren and Lawrence (KL) scale [19] and the OARSI atlas [20].

3) Magnetic resonance imaging (MRI) of the dominant hand.

MRI with gadolinium contrast enhancement is performed according to a standardised protocol at the Diakonhjemmet Hospital on the dominant hand in participants with self-reported hand OA in the population-based postal survey (Q1) and without contraindications for MRI examination. A highfield (1.0T) extremity MRI unit (ONI; GE Healthcare, Waukesha, Wisconsin, USA) is used to examine the second to fifth proximal interphalangeal (PIP) and distal interphalangeal (DIP) joints. The acquisition, MRI sequences, and scoring of MRI features (The Oslo Hand Osteoarthritis MRI score) have previously been described [83].

4) Ultrasound.

Two trained and experienced medical students perform the bilateral ultrasound examination of the hand, hip, and knee joints of all participants with supervision from an experienced sonographer/rheumatologist (HBH). A linear array transducer is used (5–13 MHz, Siemens Antares, Sonoline; Siemens Medical Solutions, Mountain View, California, USA) with fixed settings of the machine. To ensure standardisation, the same ultrasound machine without software upgrading is used throughout the study.

**Hand:** The participants sit with hands resting on a small table. Osteophytes are defined as cortical protrusions, and the presence of osteophytes is scored on a 0-3 scale as previously described [34]. In each finger joint the proximal and distal part are assessed as a whole, and the largest osteophyte determines the score independently of the number and location of other osteophytes in the same joint. The sonographers score osteophytes in the following 15 joints bilaterally (standard scanning projections): carpometacarpal (CMC) 1 (radio-palmar), metacarpophalangeal (MCP) 1–5 (dorsal), PIP 1–5 (dorsal) and DIP 2–5 (dorsal). Each joint is scanned longitudinally from the radial to the ulnar side, and transverse scanning is performed if there is uncertainty about the presence of pathology. In addition, scoring of synovitis (B-mode) and vascularity (power Doppler) (both on 0-3 scales) are performed according to previous descriptions [84].

**Hip:** The participants lie in supine position. The sonographers score the size of osteophytes at the femoral neck (score 0-3), measure the maximal capsule thickness at the femoral neck as well as evaluate the form of the capsule and the shape of the femoral head. In addition, the participants are asked about groin pain during the last week.

**Knee:** The participants lie in supine position with the knee extended for scoring of osteophyte sizes at the medial and lateral joint space (0-3 scale). In addition, meniscus protrusion medially and laterally is recorded, and the degree of synovitis in the suprapatellar recess is scored [84]. With the knee flexed to 90°, the thickness of the cartilage at the distal femur is measured in millimetres at the sulcus as well as at the lateral and medial condyle. Additionally, the cartilage quality at the distal femur is evaluated, and the presence of calcium crystal pyrophosphate deposition (CPPD) changes are recorded. All participants are asked about knee pain during the last week.

5) Functional assessment.

Physiotherapists perform the following functional assessments according to standardised protocols: Moberg Pick Up Test [85], 6 minute Walk Test [86], and 30 second Timed Stand Test [87]. Hypermobility is assessed using the Beighton scale [88], and maximal grip strength is measured using Jamar Dynamometer. The passive range of motion of the hip and knee joints is examined in supine and prone positions with the use of fixation belts.

6) Cardiovascular assessment.

Trained medical students perform the cardiovascular assessments after instructions from two experienced researchers in cardiovascular medicine (SP and AGS). Brachial blood pressure (BP) and heart rate are measured using an Omron M7 after a minimum 5 minute rest in a supine position in a quiet room. Repeated measurements are performed until two of the measurements have a ≤ 5 mmHg difference in both systolic and diastolic pressures, and a mean is calculated. A 12-lead electrocardiogram (ECG) is recorded digitally in this setting. The ankle-brachial index (ABI) is computed in the standardised fashion [89]. The systolic pressure is estimated by a Sonotrax - Pocket Doppler Vascular - 8MHz probe in the posterior tibial and dorsalis pedis arteries in both legs. The highest of these distal pressures is divided by the brachial systolic pressure to obtain the ABI. Due to time limitations the ABI is performed in every second individual.

Pulse wave analysis (PWA) using the Sphygmocor apparatus (Atcor Australia) is performed to estimate the pulse wave velocity (PWV) and augmentation index (AIx). We have chosen to estimate the carotid-femoral PWV (cfPWV) between the sites on the carotid and on the femoral artery where the pulse is most strongly palpated. The start of the pressure waves at the carotid and femoral artery are ECG gated to adjust for transit time. The PWV is calculated from knowing both the transit-time for the pulse wave travelling from the heart to the two sites and the distance between these sites [90]. The AIx is defined as the change in pressure between the second and first systolic peaks as a percentage of the pulse pressure and is standardised to a heart rate of 75. AIx is calculated by applying a validated transfer system to pressure recordings of the arterial pressure waves at the radial artery [91]. Individuals suffering from atrial fibrillation are excluded from the PWA analysis. Based on prior studies, the participants are requested to abstain from food, drinks (except for water), and smoking for at least 3 hours prior to the PWA examinations [90].

7) Body weight and height, hip and waist circumference, and digital photo of hands.

The weight and height are measured on all participants with shoes removed and pockets emptied. The hip circumference is measured using a tape measure around the maximum circumference, and waist circumference is measured at the narrowest waist after relaxed exhalation. A standardised digital photo from above, with both hands on top of a black fabric on a small tray, is taken by the medical student performing the ultrasound examination.

8) Blood and urine sample.

Blood (full blood and sera) and urine samples are collected and stored in a biobank for future analyses of associations between clinical characteristics and biomarkers or candidate genes. Blood samples are analysed for haemoglobin and erythrocyte sedimentation rate at the hospital laboratory.

**Table 3:** Questions to complete during the clinical examination (Q3)

**Measure**	**Measurement scale**
Pain/stiffness in or around the joint on most days in the precious month	Yes/No
Average joint pain the past week	NRS: 0-10
Which joint most painful	Hip/Knee/Hand
Fatigue past week	NRS: 0-10
Patient global assessment of the OA disease	NRS: 0-10

**Table 4:** Measures included in the comprehensive clinical examination (CE)

**Measure**	**Measurement scale**
**Medical examination**	
Lenght of time with OA disease	Year of OA diagnosis
Lenght of time with OA symptoms	Year when first experienced OA symptoms
Dominant hand	Right/left
Dominant leg	Right/left
Father with OA	Yes/No
Mother with OA	Yes/No
Siblings with OA	Yes/No
Tegner activity scale [81]	0-5
Stroke test [80]	0-3
Soft tissue swelling joint count (SJC)	Shoulder, acromioclavicular (AC), elbow, wrist, CMC, MCP, PIP, DIP, hip, knee, ankle, metatarsophalangeal (MTP); Yes/No
Bony enlargement joint count (BEJC)	Shoulder, AC, elbow, wrist, CMC, MCP, PIP, DIP, hip, knee, ankle, MTP; Yes/No
Tender joint count (TJC)	Shoulder, AC, elbow, wrist, CMC, MCP, PIP, DIP, hip, knee, ankle, MTP+ cervical columna, thoracal columna, lumbal colulma; Yes/No
Limited motion joint count (LMJC)	Shoulder, AC, elbow, wrist, CMC, MCP, PIP, DIP, hip, knee, ankle, MTP+ cervical columna, thoracal columna, lumbal colulma; Yes/No
ACR classification criteria for hip OA [17]	Hip pain AND at least 2 of the following 3 features: erythrocyte sedimentation rate (ESR) <20mm/hour, radiographic femoral or acetabular osteophytes, radiographic joint space narrowing
ACR clinical classification criteria for knee OA [16]	Knee pain AND at least 3 of 6 of the following features: age > 50, stiffness < 30 min, crepitus, bony tenderness, bony enlargement, no palpable warmth
ACR classification criteria for hand OA [18]	Hand pain, aching or stiffness AND 3 or 5 of the following features: hard tissue enlargement of ≥2 of 10 selected joints, hard tissue enlargement of ≥2 DIP joints, fewer than 3 swollen MCP joints, deformity of ≥1 of 10 selected joints (bilateral 2^nd^-3^rd^ DIP, 2^nd^-3^rd^ PIP, and 1^st^ CMC)
Doctor global assessment of OA disease	NRS: 0-10
**Physical function examination**	
Range of movement	Hip: flexion, extension, abduction, internal rotation, external rotation
	Knee: flexion, extension
Beighton scale [88]	0-9
Maximum grip strenght (JAMAR dynamometer)	Kilograms (mean of 3 repetitions)
Moberg Pick Up Test [85]	Seconds (Left/Right hand)
30 sec Timed Stand Stands [87]	Number of stands
6 Minutes Walk Test [86]	Meters
Leg pain during 6 Minutes Walk Test	Yes/No + NRS: 0-10
**Imaging**	
**Magnetic resonance imaging**	Synovitis grade 0-3, flexor tenosynovitis grade 0-3, erosion grade 0-3, cyst absent/present, osteophyte grade 0-3, joint space narrowing grade 0-3, malalignment absent/present, bone marrow lesion grade 0-3, collateral ligament pathology absent/present (the Oslo hand OA MRI score)
Dominant hand (only when self-reported hand OA)	
**Conventional radiographs**	
Hip joints, bilateral	Millimetre joint space width
Knee joints, bilateral	Kellgren and Lawrence scale: grade 0-4
	OARSI: medial and lateral femoral condyle/medial and lateral tibial plateau osteophyte grade 0-3+, medial and lateral compartment joint space narrowing grade 0-3+, medial tibial attrition absent/present, medial tibial sclerosis absent/present, lateral femoral sclerosis absent/present
Hand joints, bilateral	Kellgren and Lawrence scale: grade 0-4
	OARSI: osteophyte grade 0-3, joint space narrowing grade 0-3, malalignment absent/present, erosion absent/present sclerosis absent/present, cyst absent/present
**Ultrasound**	
HIP joint osteophytes	0, none; 1, minor; 2, moderate; 3, major size of osteophytes
Capsule diameter caput	Millimetre
Capsule diameter collum	Millimetre
Capsule shape	0, concave; 1, flat; 2, convex
Caput femoris shape	0, normal; 1, minor flattening; 2, considerably changed shape
Groin pain last week	0, no pain; 1, some pain; 2, moderate pain; 3, severe pain
KNEE joint osteophytes, femur medial and lateral	0, none; 1, minor; 2, moderate; 3, major size of osteophytes
Knee joint osteophytes, tibia medial and lateral	0, none; 1, minor; 2, moderate; 3, major size of osteophytes
Lateral and medial menisci protrusion	0, none; 1, minor; 2, major
Synovitis in the suprapatellar recess (combined synovitis and effusion)	0, none; 1, minor; 2, moderate; 3, major
Femur cartilage height at sulcus, lateral, and medial condyle	Milimeter
Femur cartilage quality, Degree of hyperechogenisity	0, normal; 1 minor; 2 major
Femur hyaline cartilage, Calcium crystal pyrophosphate deposition (CPPD)	0, none; 1, presence of CPPD changes
Kne pain last week	0, no pain; 1, some pain; 2, moderate pain; 3, severe pain
HAND joint osteophytes	30 joints: 0, none; 1, minor; 2, moderate; 3, major size of osteophytes
Hand joint synovitis (combined synovitis and effusion)	30 joints: 0, none; 1, minor; 2, moderate; 3, major
Hand joint vascularity	30 joints: 0, none; 1, minor (1-2 small vessels); 2, moderate; 3, major
**Cardiovascular examination**	
Heart rate	Beats per minute
Systolic blood pressure (monitor)	mmHg
Diastolic blood pressure (monitor)	mmHg
Ankle brachial index (ABI)	Systolic blood pressure leg (mmHg)/ Systolic blood pressure arm (mmHg)
Arterial stiffness	Pulse wave velocity, augmentation index
ECG	Electrocardiogram
**Surgery/injuries**	
Hip prosthesis	Yes/No; Right/Left; Year of surgery
Hip surgery/injury	Yes/No; Right/Left; Type surgery/injury; Type treatment; MD/Hospital
Knee prosthesis	Yes/No; Right/Left; Year of surgery
Knee surgery/injury	Yes/No; Right/Left; Type surgery/injury; Type treatment; MD/Hospital
Hand surgery/injury	Yes/No; Right/Left; Type surgery/injury; Type treatment; MD/Hospital
Hand synovectomy	Yes/No; Number of operations
Hand joint prosthesis	Yes/No; Number of operations
Hand joint fusion	Yes/No; Number of operations
Other operations on hand joints or tendons	Yes/No; Number of operations
**Anthropometric data**	
Body weight	Centimeters
Body height	Kilograms
Hip circumference	Centimeters
Waist circumference	Centimeters
**Digital photo of hands**	Standardised photo from above, both hands on top of a black fabric on a small tray
**Biological samples**	
Blood haemoglobin	g/dL
Blood erythrocyte sedimentation rate (ESR)	mm/h, whole blood and blood serum are stored in a biobank
Urine	Are stored in a biobank

#### Data from a previous study in Ullensaker Municipality

The Ullensaker Study is a population based epidemiological cohort study with musculoskeletal pain as the primary focus and with three preceding surveys conducted in 1990, 1994, and 2004 [92-95]. The surveys were sent to all inhabitants in the Ullensaker Municipality in the following birth cohorts: 1918-20, 1928-30, 1938-40, 1948-50, 1958-60, 1968-70, and 1978-80 (only in 2004). Hence, for a subsample, we will be able to merge data to the previously collected data in The Ullensaker Study on musculoskeletal pain and functional ability in addition to demographic factors, body height and weight, comorbidity, mental distress, sleep, sick leave, health care utilisation, and physical activity. We will investigate if any of these previous data can be identified as predictors of incident OA.

#### Data from other sources

Along with the initial postal questionnaire, the participants are asked for consents to merge the data to national data registries (e.g. The Norwegian Arthroplasty Register, The register of The Norwegian Labour and Welfare Administration, The Norwegian Prescription Database, The Medical Birth Registry of Norway, The Cancer Registry of Norway, and The Norwegian Cause of Death Registry). Furthermore, those who have previously participated in Ullensaker Study are asked for permission to merge with the data collected in 1990, 1994, and/or 2004.

#### Project timeline

**Figure 1:**
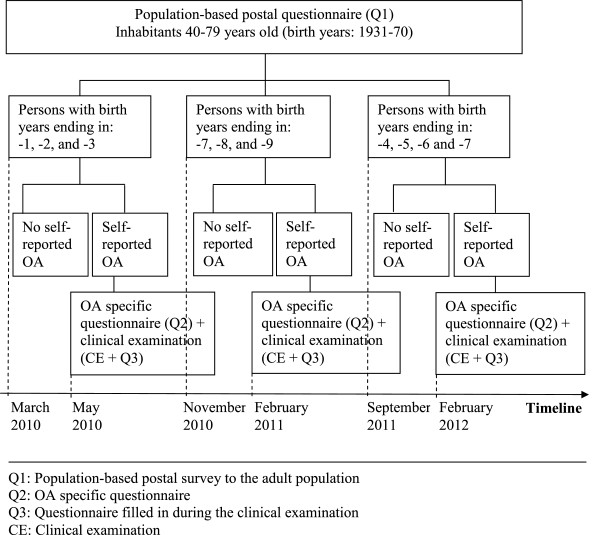
Study timeline.

In order to avoid a large time lag between the initial population-based postal survey questionnaire (Q1) and the subsequent clinical examination (Q2 and CE), the Q1 questionnaire are sent in three dispatches to allow time for the clinical examinations in the interim (Figure 1). Dispatch no.1 targets inhabitants with birth year ending in 1, 2, or 3 with the questionnaires being sent by mail in March 2010. The next dispatch approaches the previous Ullensaker Study participants (birth years ending in 8, 9, or 0) and is mailed in November 2010, which is the same time of the year as the three preceding surveys. The questionnaires for the third dispatch are sent in September 2011 to inhabitants with birth years ending in 4, 5, 6, or 7. We estimate that the clinical examination for the third dispatch will be completed in the spring 2013.

#### Statistical analyses

Parametric and non-parametric statistical analysis models will be used depending on the distribution of the variables.

#### Sample size

The response rates in the preceding Ullensaker Study surveys decreased from 67% in 1990, to 63% and 55% in 1994 and 2004, respectively. The results from 2004 showed an overall self-reported OA prevalence of 12.8% in the total sample aged 25 to 75 years [10]. In January 2010, the total population in Ullensaker Municipality was approximately 29 000 persons, and our estimations suggested that the target adult population is about 12 000 persons.

#### Ethical aspects

The study was approved by the Norwegian Regional Committee for Medical and Health Research Ethics (Ref.no: 2009/812a (population-based survey questionnaire (Q1)) and 2009/1703a (OA specific questionnaires and clinical examination (CE, Q2 and Q3)) and the Norwegian Data Protection Authority. The study sample receives written information about the study, and separate written consents are given for the initial baseline questionnaire and for the clinical examination.

## Discussion

This population-based observational study will provide important knowledge about the prevalence of symptomatic and radiographically confirmed OA, risk factors, consequences of OA, comorbidity in persons with OA, and quality of OA care in Norway. Outcomes may be linked to prevalence estimates of study sample subsets according to radiographic classification of OA severity, as has previously been performed with rheumatoid arthritis [96].

A major advantage in this study is the opportunity to merge the data with other data sources. Data will be merged with data from the previous Ullensaker study, national registries, and with the data from the comprehensive clinical examination, which include several imaging techniques, patient-reported outcome measurements, performance-based tests, cardiovascular morbidity, and data on metabolic syndrome. Hence, a wide spectre of analyses may be done on the merged data. Furthermore, more research on the association between self-reported hand, hip, and knee OA and clinically or radiologically confirmed OA will provide valuable knowledge for future epidemiological studies on OA.

The value of population-based studies is however related to the response rate. In the previous Ullensaker Study surveys the response rate has shown to decrease over time, which is in line with other populations studies showing a decrease in participation rates during the past three to four decades [97]. Whether a poor participation rate will be a problem, is related to the representativeness of the participants since low participation can provide more opportunity for bias to occur. Our opportunities to explore potential selection bias are limited, but we will be able to compare participants and non-participants in relation to age and gender as well as compare participants with regional or national mean frequencies for other demographic factors (i.e. marital status, education, and employment status).

There are plans for future follow-up surveys for the MUST participants and clinical examinations of the MUST OA cohort, which will provide further knowledge about various factors, for example about trends in OA prevalence, prognosis according to functional ability, quality of care in health services, and health care utilisation. Hence, this population-based observational study will constitute a unique platform with a large potential for future OA research by providing valuable opportunities for various epidemiological and clinical studies on OA in Norway.

## Abbreviations

ABI: Ankle-brachial index; AC: Acromioclavicular; ACR: American College of Rheumatology; Aix: Augmentation index; AUCAN: Australian/Canadian osteoarthritis hand index; BEJC: Bony enlargement joint count; BP: Blood pressure; cfPWV: Carotid-femoral pulse wave velocity; CMC: Carpometacarpal; CPPD: Calcium crystal pyrophosphate deposition; DIP: Distal interphalangeal; ESR: Erythrocyte sedimentation rate; FIHOA: Functional index for hand osteoarthritis; HOOS: Hip disability and osteoarthritis outcome score; IPAQ: International physical activity questionnaire; KL: Kellgren and Lawrence scale; KOOS: Knee injury and osteoarthritis outcome score; LMJC: Limited motion joint count; MCP: Metacarpophalangeal; MDHAQ: Multidimensional health assessment questionnaire; MHOQ: Michigan hand outcomes questionnaire; MTP: Metatarsophalangeal; MUST: Musculoskeletal pain in Ullensaker study; MRI: Magnetic resonance imaging; NRS: Numeric rating scale; OARSI: Osteoarthritis research society international; OA: Osteoarthritis; OA-QI: Osteoarthritis quality indicator questionnaire; PIP: Proximal interphalangeal; PWA: Pulse wave analysis; PWV: Pulse wave velocity; SJC: Soft tissue swelling joint count; SNQ: Standardised Nordic questionnaire; TJC: Tender joint count; US: United States

## Competing interests

The authors declare that they have no competing interests.

## Authors’ contributions

All authors participated in the design of the study. NØ drafted this article, but all authors were involved in drafting the article or revising it critically for important intellectual content. All the authors have read and approved the final manuscript.

## Pre-publication history

The pre-publication history for this paper can be accessed here:

http://www.biomedcentral.com/1471-2474/14/201/prepub
